# Physician Perceptions of Cannabidiol (CBD) and Cannabis in Sports Medicine and Performance

**DOI:** 10.1155/2023/8824466

**Published:** 2023-12-11

**Authors:** Brendon Ross, Thomas Trojian, Daniel M. Cushman

**Affiliations:** ^1^University of Chicago, Department of Orthopedic Surgery & Rehabilitation Medicine, Chicago, IL, USA; ^2^Temple University Student Health, Philadelphia, PA, USA; ^3^University of Utah, Department of Physical Medicine & Rehabilitation and Department of Orthopaedics, Salt Lake City, UT, USA

## Abstract

**Objectives:**

There is growing evidence regarding cannabinoid use in sports medicine and performance, especially cannabidiol (CBD). This study aims to determine if sports medicine physicians are recommending cannabinoids for therapeutic purposes, as well as analyze perceptions of cannabinoids within sports medicine and performance.

**Methods:**

Physician members of the American Medical Society for Sports Medicine (AMSSM) completed an anonymous survey on demographics, CBD and Cannabis recommendations, as well as attitudes toward cannabinoid products within sports medicine. Factors associated with CBD and cannabis recommendations as well as perceptual differences were found using multivariate regression modelling.

**Results:**

Responses from 333 physicians were recorded. The following groups were less likely to agree with allowing cannabis for recreational purposes: female gender (coeff. = 0.79 (0.33–1.25), *p*=0.001), increasing age (coeff. = 0.04 (0.02, 0.07), *p*  <  0.001), and rural respondents (compared to baseline urban, coeff. = 1.16 (0.36, 1.95), *p*=0.004). Similarly, these three factors were associated with a higher likelihood of disagreeing with WADA removing cannabis from the prohibited substance list and with the NCAA allowing CBD use by collegiate athletes (*p* ≤ 0.045). CBD was less likely to be recommended by pediatricians, rural physicians, and academic physicians (*p* ≤ 0.030). Male physicians and younger physicians were less likely to identify cannabis as performance-enhancing (*p* ≤ 0.042).

**Conclusions:**

Sports medicine physicians have varying views on cannabinoids. While sports medicine physicians generally have favorable attitudes toward CBD and cannabis, these perceptions appear to be significantly affected by age, practice type, and gender.

## 1. Introduction

Over the past decade, the United States (US) has seen increased growth in the interest and use of cannabis and cannabidiol (CBD) for therapeutic purposes [1–3]. Cannabis has been studied for its therapeutic role in chronic pain disorders and cancer-related pain. Epidiolex (r) (cannabidiol, Jazz Pharmaceutical) is a recent FDA-approved form of CBD, which has been shown to reduce seizure frequency in certain types of seizure disorders [4, 5]. In addition, there is emerging evidence about the potential applications of cannabinoids within sports medicine, especially CBD. Unlike tetrahydrocannabinol (THC), CBD lacks side effects and is safe and well-tolerated in human studies [4, 6–8].

CBD in the health and wellness industry has propelled the growth of cannabinoid use, with the total global CBD market estimated to reach 47.22 billion by 2028, up from 4.9 billion in 2021 [9]. In addition, there is growing evidence that CBD has increased significantly among athletes in elite sports [10, 11]. From Mike Tyson to Megan Rapinoe, each with their own line of CBD products, more professional athletes seek to legitimize CBD use in sports. This shift may be related to the removal of CBD from the World Anti-Doping Agency (WADA) prohibited substance list in 2018, in addition to the evolving legal landscape and marketing across the US [12].

Because of this evolution in sports medicine, physicians may be experiencing more inquiries about these products in the clinic or training room. While opinions regarding cannabinoids have been examined in other areas of medicine, including psychiatry, family medicine, and neurology, evidence is currently sparse within the field of sports medicine [13–16]. This survey study aims to determine if sports medicine physicians are recommending CBD and cannabis products for therapeutic purposes and to analyze attitudes and perceptions of CBD and cannabis within sports medicine.

## 2. Methods

Physician members of the American Medical Society for Sports Medicine (AMSSM) received an emailed survey on two separate occasions, the second email being a reminder email to complete the survey in January and March 2022. An estimated 4,910 emails were sent, and 3,079 (62.7%) were opened. Utilizing prior studies as a reference for the anonymous survey, we included a variety of questions on demographic information as well as attitudes toward CBD and cannabis products [13, 15–17]. Definitions of CBD, THC, and cannabis were provided at the beginning of the survey for reference (CBD = Cannabidiol, this is the nonpsychoactive cannabinoid found in the hemp plant that is marketed in various products which are legal in 47 states (except Idaho, South Dakota, and Nebraska) if the THC content is less than 0.3%; THC = Tetrahydrocannabinol. This is the main psychoactive cannabinoid of cannabis; cannabis = also known as marijuana, these products contain various levels of cannabinoids including THC and CBD. Data were stored on REDCap [18]. Statistical analysis was performed with Stata 17.0 (StataCorp LLC, College Station, TX).

Frequencies and percentages evaluate demographic data. Likert scale variables included strongly agree (1), agree (2), neutral (3), disagree (4), and strongly disagree (5); numbers 1 and 2 and numbers 4 and 5 were combined only for display purposes in tables. Logistic and ordinal regression models (using the full Likert scale) were used to compare dependent variables to independent variables (sex (categorical male/female), age (numeric), practice location (categorical, reference urban), practice setting (categorical, reference academic), team physician (categorical yes/no), primary specialty (categorical, reference family medicine), and legality within the state (categorical yes/no)). Reference values were set to the most common value within the variable. *P* values were set at ≤0.05 for significance.

## 3. Results

**Figure 1:**
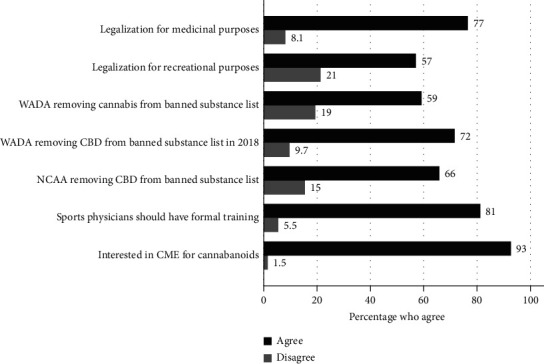
Physician attitudes and perceptions of cannabinoids. Survey results. Dark line denotes percentage of respondents who agree with the following statements. Respondents were allowed to choose a neutral category (neither agree nor disagree, not pictured). CBD = cannabidiol; CME = continuing medical education.

A total of 333 responses were completed (10.8% response rate of opened emails), with a mean age 42 (SD 10.3) years.  Table 1 outlines the demographic and practice-related information of respondents.  Figure 1 describes the views of respondents regarding CBD and cannabis. The following groups were less likely to agree with allowing cannabis for recreational purposes: female gender (coeff. = 0.79 [0.33–1.25], *p*=0.001), increasing age (coeff. = 0.04 [0.02, 0.07], *p*  <  0.001), and rural respondents (compared to baseline urban, coeff. = 1.16 [0.36, 1.95], *p*=0.004). Similarly, these three factors were significantly associated with a higher likelihood of disagreeing with WADA removing cannabis from the prohibited substance list (female gender coeff = 0.64 [0.18–1.11], *p*=0.007, increasing age coeff. = 0.06 [0.03–0.08], *p*  <  0.001, and rural coeff. = 1.17 [0.39–1.94], *p*=0.003). In addition, these three factors (female, rural, and increasing age) were not in agreement with the NCAA (National Collegiate Athletic Association) permitting CBD use by collegiate athletes (female gender (coeff 0.64–0.79, *p*  < =0.008); rural location when compared to urban (coeff 0.84–1.17, *p*  < =0.045; and increasing age (coeff 0.045–0.056, *p*  <  0.001)].

### 3.1. CBD and Cannabis Recommendations

**Figure 2:**
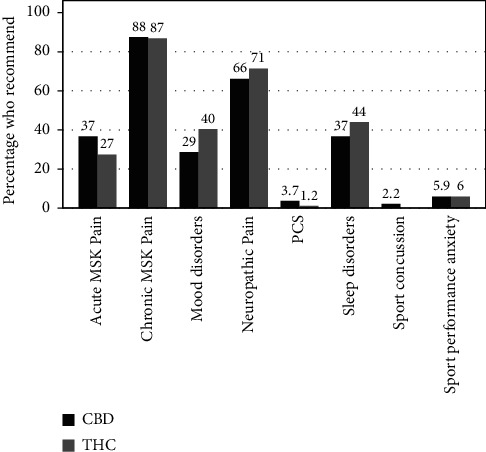
Physician recommendations of CBD and cannabis. Survey results. *Y*-axis refers to percentage of respondents who recommend each substance for the conditions listed on the *x*-axis. CBD = cannabidiol. THC = tetrahydrocannabinol. MSK = musculoskeletal; PCS = postconcussive syndrome.

40.8% of survey respondents have recommended CBD products for therapeutic purposes, compared to 24.8% of respondents having recommended cannabis products.  Figure 2 represents indications for CBD and cannabis recommendations among respondents. Regression analysis revealed that community/private practice physicians were more likely to recommend CBD compared to academic physicians (OR = 1.76 [1.05–2.95], *p*=0.030), as well as being more likely to recommend Cannabis (OR 2.02 [1.10, 3.71], *p*=0.023). Pediatricians were less likely to recommend CBD compared to family medicine (OR 0.28 [0.10–0.79], *p*=0.016). Rural physicians were less likely to recommend cannabis compared to urban physicians (OR 0.23 [0.06, 0.86], *p*=0.029). Lastly, no pediatricians recommended cannabis.

### 3.2. Cannabinoids and Sports Performance

**Figure 3:**
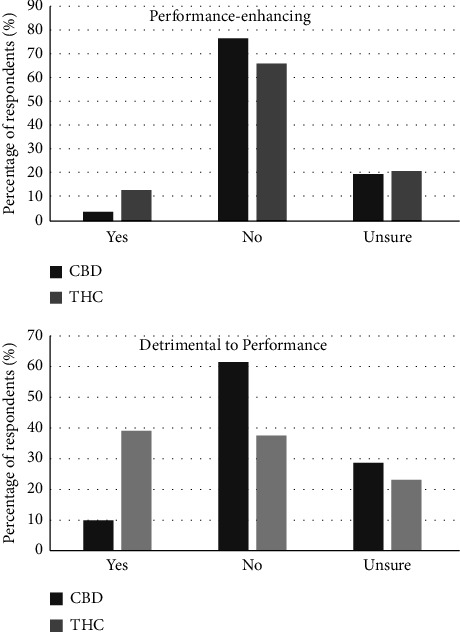
Physician perceptions of cannabinoids and sports performance. Survey results. Percentage of respondents who feel that each substance is performance-enhancing or detrimental to performance. CBD = cannabidiol. THC = tetrahydrocannabinol.

Figure 3 illustrates the perceptions of respondents about cannabinoids and sports performance. Older respondents were more likely to name CBD as detrimental to performance and the integrity of sport (OR = 1.05 [1.01–1.09], *p*=0.018). Similarly, older respondents were more likely to view cannabis as detrimental to performance and the integrity of sport (OR = 1.04 [1.01–1.07], *p*=0.007). Male physicians were less likely to identify cannabis as performance-enhancing (OR 0.38 [0.18, 0.82], *p*=0.013).

## 4. Discussion

The medicinal use of cannabis has been recorded for millennia, dating back to Emperor Shen Neng of China in 2737 BCE. In 1993, the mummy of the Princess of Ukok, better known as the Siberian Ice Maiden, was discovered by Russian archaeologists in Southern Siberia. Along with her 2500-year-old remains, a pouch of cannabis was found, which scientists speculate was used to cope with her symptoms of breast cancer and other ailments [19]. While more than 100 different cannabinoids within the marijuana plant contribute to its therapeutic potential, THC and CBD remain the most well-studied [4, 8, 20].

THC is the primary psychoactive cannabinoid acting as a partial agonist of CB1 and CB2 receptors of the endocannabinoid system (ECS). CBD acts as a primary allosteric modulator of CB1 and CB2 receptors, enhancing the activity of the endogenous ECS; however, CBD is reported to be devoid of psychoactive effects [8, 21, 22]. This allosteric modulation by CBD has also been shown to inhibit THC-elicited psychotomimetic potential and cognitive impairments [21, 23, 24]. Considering emerging evidence of CBD's potential analgesic, anxiolytic, and neuroprotective effects, its lack of intoxicating effects makes it ideal for medicinal use [8].

Recent physician survey studies have conveyed growing acceptance among various specialties about the recreational and therapeutic uses of CBD and cannabis [13–17]. Respondents in our study appear to have generally favorable attitudes towards CBD and cannabis, with most respondents favoring the legalization of medicinal and recreational cannabis. More interestingly, this study demonstrates that sports medicine providers recommend CBD and cannabis products for various therapeutic purposes, primarily chronic musculoskeletal and neuropathic pain. Over the last few decades, there has been ample medical literature demonstrating improvements in symptoms related to chronic musculoskeletal and neurologic conditions using CBD and cannabis (i.e., fibromyalgia, spasticity, cancer-related pain) [25–27]. For example, a recent survey study of 878 patients with fibromyalgia reported that 72% of respondents substituted CBD for pain medications, mainly opioids or benzodiazepines, primarily substituting for harm reduction (i.e., fewer side effects) [28]. Similarly, in 2017, 595 Parkinson's disease and multiple sclerosis patients were surveyed, and 44% reported cannabis use for therapeutic purposes with high efficacy and lowered self-reported disability and prescription drug use [29]. More providers within our study recommended CBD compared to cannabis, 40.8% vs 24.8%. The reasons are not entirely clear from this study, but given the overall safety profile of CBD, its lack of “intoxicating” effects, and the general infiltration of CBD into mainstream consumer products, providers may see CBD as a safer option for patients compared to Cannabis and THC-containing products [30].

When we examined the factors potentially influencing CBD and cannabis recommendations of survey respondents, the community/private practice setting had a higher likelihood of recommending CBD and cannabis products for therapeutic purposes than the academic setting. In addition, respondents practicing in rural locations were much less likely to recommend cannabis products than those practicing in urban locations. One could speculate over an exhaustive list of socioeconomic and political factors which may play into these findings, but it is unclear from this study. The most important aspect of these findings is that other expected factors, notably age, gender, and primary specialty (apart from pediatrics), did not appear to influence the CBD or cannabis recommendations of survey respondents. Why is this important? Perhaps this reflects a growing acceptance of cannabis and CBD concerning their more evidence-based applications among providers; however, when examining perceptions of cannabinoids and sports performance, exciting age and gender differences were observed within our study.

Recent cases involving elite athletes have brought renewed attention to the debate over cannabis and whether it should be considered “doping” in sport. There is inconclusive evidence about any direct performance-enhancing effects of cannabis or CBD in athletes [31]. Despite WADA removing CBD from the prohibited substance list in 2018, cannabis remains prohibited in competition by WADA and many other professionals and international organizations. Most respondents in our study favored WADA removing cannabis from the prohibited substance list; however, older female providers were significantly less likely to favor this decision. Interestingly, to account for these age and gender differences, we found that male respondents were significantly less likely to view cannabis as performance-enhancing. In addition, older respondents were more likely to view cannabis and CBD as detrimental to performance and the integrity of sports. When looking closer at these perceptual differences, we saw an interesting distinction between CBD and cannabis.

Following the shift from being a prohibited to an allowed substance, CBD has quickly become a booming multibillion-dollar industry in sports. Several professional athletes have new lines of CBD products marketed for various claims, including pain relief, improvements in sports performance anxiety, and postexercise recovery [6, 20]. Leas et al. revealed that in April 2019, Google searches for CBD surpassed acupuncture by a factor of 7.49, meditation by 3.38, exercise by 1.59, and marijuana by 1.13 with 6.4 million searches [2]. Has this surge influenced sports medicine providers to think differently about CBD in sports? While older respondents in our survey were significantly more likely to think CBD is detrimental to performance, only 9.9% of our respondents believe CBD is detrimental to performance and the integrity of sport, compared to 39% with cannabis. The reasons for this discrepancy are unclear from this study, but these perceptions may influence how sports medicine providers counsel their athletes using CBD products. It is important to note that the ergogenic versus ergolytic effects of CBD compared to cannabis are still largely unknown; therefore, these perceptual differences can largely, if not exclusively, be attributed to marketing and advertising. In addition, one must recognize the seemingly ubiquitous addition of CBD to countless consumer products, which may also contribute to this evolving distinction.

There are several limitations to this study. First, the study design is only one data point; therefore, the overall trends of CBD and cannabis recommendations by sports medicine providers increasing or decreasing in the current culture are uncertain. It would be interesting to reevaluate these questions in five to ten years to observe any trends given the evolving legal landscape, social acceptance, and relative integration of CBD and cannabis into mainstream culture. The small sample size accounts for approximately 7% of AMSSM membership. However, the demographic distributions of our survey follow very closely with membership distributions (i.e., 71% of AMSSM have family medicine as their primary specialty, with our survey having 69% family medicine respondents). Lastly, although the survey was anonymous, this is still considered a fringe topic by many in sports medicine and medicine in general, which may limit the divulgence of actual behaviors and attitudes of respondents. Regardless of these limitations, 98% of respondents in our survey agreed to learn more through continuing education, recognizing that these cultural shifts are certainly not going away.

## 5. Conclusion

This study reveals that sports medicine providers generally have favorable attitudes toward CBD and cannabis, but these perceptions appear to be affected by age and gender. Many sports medicine providers are recommending CBD and cannabis products. They mainly recommend it for chronic musculoskeletal and neuropathic pain. However, this is the first study to reveal that providers, albeit very few, are also recommending these products for sport-related concussions and sports performance anxiety. Most sports medicine providers favor the removal of cannabis from the prohibited substance list of WADA; however, we observed significant differences regarding the perceived effects of CBD and cannabis on sports performance among providers. This advancing cultural shift motivates ongoing research and education for sports medicine providers to better answer questions posed by athletes about the safety, dosing, and potential effects of CBD and cannabis in sports.

## Figures and Tables

**Table 1:** Demographic information for survey respondents. *N* = 333.

	*n*	(%)
Female sex	103	30.8
Specialty		
Emergency medicine	13	3.9
Family medicine	231	69.4
Internal medicine	24	7.2
Pediatrics	29	8.7
Physical medicine and rehabilitation	28	8.4
Other	8	2.4
Practice location		
Rural	28	8.4
Suburban	140	42.2
Urban	164	49.4
Practice setting		
Academic/university	195	58.9
Community/private practice	131	39.6
Veteran affairs/government	5	1.5
Team physician	246	74.5
High school	173	52.4
College	159	48.2
Professional	70	21.2
Olympic	24	7.3
Paralympic	5	1.5
Other	23	7.0

## Data Availability

Supporting data can be located on REDCap, a secure online platform for building surveys and databases.
